# USP17 mediates macrophage-promoted inflammation and stemness in lung cancer cells by regulating TRAF2/TRAF3 complex formation

**DOI:** 10.1038/s41388-018-0411-0

**Published:** 2018-07-23

**Authors:** Chih-Hao Lu, Da-Wei Yeh, Chao-Yang Lai, Yi-Ling Liu, Li-Rung Huang, Alan Yueh-Luen Lee, S.-L. Catherine Jin, Tsung-Hsien Chuang

**Affiliations:** 1 Immunology Research Center, National Health Research Institutes, Miaoli, Taiwan; 2 Department of Life Sciences, National Central University, Zhongli District, Taoyuan City, Taiwan; 3 Institute of Molecular and Genomic Medicine, National Health Research Institutes, Miaoli, Taiwan; 4 National Institute of Cancer Research, National Health Research Institutes, Miaoli, Taiwan; 5 Program in Environmental and Occupational Medicine, Kaohsiung Medical University, Kaohsiung, Taiwan

## Abstract

Macrophage accumulation and inflammation in the lung owing to stresses and diseases is a cause of lung cancer development. However, molecular mechanisms underlying the interaction between macrophages and cancer cells, which drive inflammation and stemness in cancers, are poorly understood. In this study, we investigated the expression of ubiquitin-specific peptidase 17 (USP17) in lung cancers, and role of elevated USP17 in the interaction between macrophages and lung cancer cells. USP17 expression in lung cancers was associated with poor prognosis, macrophage, and inflammatory marker expressions. Macrophages promoted USP17 expression in cancer cells. TNFR-associated factor (TRAF) 2-binding and TRAF3-binding motifs were identified in USP17, through which it interacted with and disrupted the TRAF2/TRAF3 complex. This stabilized its client proteins, enhanced inflammation and stemness in cancer cells, and promoted macrophage recruitment. In different animal studies, co-injection of macrophages with cancer cells promoted USP17 expression in tumors and tumor growth. Conversely, depletion of macrophages in host animals by clodronate liposomes reduced USP17 expression and tumor growth. In addition, overexpression of USP17 in cancer cells promoted tumor growth and inflammation-associated and stemness-associated gene expressions in tumors. These results suggested that USP17 drives a positive-feedback interaction between macrophages and cancer cells to enhance inflammation and stemness in cancer cells, and promotes lung cancer growth.

## Introduction

Lung cancer is the most commonly diagnosed cancer and the leading cause of cancer-related death worldwide. The two main histological subtypes are non-small-cell lung cancer and small-cell lung cancer, accounting for 85% and 15% of cases, respectively [1, 2]. Inflammatory stress is a major risk factor for lung cancer. The tumor microenvironment contains various cells, including cancer cells, cancer stem cells (CSCs), and stromal cells such as fibroblasts, endothelial cells, and leukocytes. Most leukocytes in tumors are macrophages. These tumor-associated macrophages (TAMs) promote tumor-associated inflammation, CSC niches, and all aspects of tumor initiation, growth, and development [3–5]. In lung and other cancers, extensive macrophage infiltration is often associated with poor prognosis [6–8].

Inflammation is a hallmark of cancer development [9]. Chronic inflammation resulting from viral infections, pneumonia or tuberculosis, or chronic obstructive pulmonary disease is associated with lung cancer development. Cigarette smoking and inhaled asbestos or silica act as carcinogens by initiating chronic inflammation [6, 10–12]. Toll-like receptor (TLR), tumor necrosis factor receptor (TNFR), and interleukin (IL)-1 receptor initiate inflammatory signaling cascades in tumor cells in response to endogenous and exogenous carcinogenic stimuli, leading to nuclear factor-κB (NF-κB) activation. NF-κB regulates gene expressions involved in inflammation, anti-apoptosis, angiogenesis, and boost the proliferation, survival, and invasion of cancer cells to support tumor progression [13–15]. Inflammation also results in increased stemness-associated gene expressions, leading cancer cells to adopt a CSC phenotype [16–18]. CSCs can self-renew and differentiate to promote tumor progression and metastasis and are responsible for treatment resistance and recurrence [19, 20]. Chemotherapy remains the standard treatment for lung cancers; however, although conventional cytotoxic therapies eliminate the bulk of tumor cells, among residual cancer cells, CSCs continue to proliferate and survive [21, 22].

A total of seven TNFR-associated factor (TRAF) members (TRAF1 to TRAF7) have been characterized. These TRAFs were originally identified as adaptor proteins in the assembly of receptor-associated complexes for the regulation of signal transductions. For example, binding of TRAF2 to TNFR induces signaling, leading to the activation of NF-κB and MAPKs for the regulation of inflammatory responses and cell death and survival. These TRAFs, with the exception of TRAF1, contain an N-terminal RING finger domain known to mediate the catalytic activity of an E3 ubiquitin ligase [23–26]. For example, TRAF2 and TRAF3 promote K63-linked ubiquitination during protein–protein interactions for signal transduction [23, 24]. Furthermore, they form a complex with the cellular inhibitor of apoptotic protein (cIAP) 1 and cIAP2 to promote K48-linked ubiquitination and proteolytic degradation of client proteins [25]. Thus, depending on their target protein, TRAFs can be a positive regulator or a negative regulator in inflammatory signaling pathways.

Ubiquitination of a target molecule is a reversible process and can be counteracted by deubiquitinases. Ubiquitin-specific peptidases (USPs) comprise the largest family of deubiquitinases. Of them, the USP17 (also termed DUB3) is a member of the cytokine-inducible deubiquitinase family, which consists of USP36 (DUB1) and USP17lc (DUB2) [27, 28]. In this study, we found that high USP17 expression was associated with expression of inflammatory mediators, macrophage markers, and poor prognosis of lung cancer. Macrophages induced the expression of USP17 in cancer cells. The role and underlying mechanism of USP17 in a positive-feedback interaction between macrophages and cancer cells to promote inflammation, stemness, and progression of lung cancers were investigated.

## Results

### High USP17 expression correlate with inflammatory and macrophage marker expressions, and poor prognosis in lung cancer

**Fig. 1:**
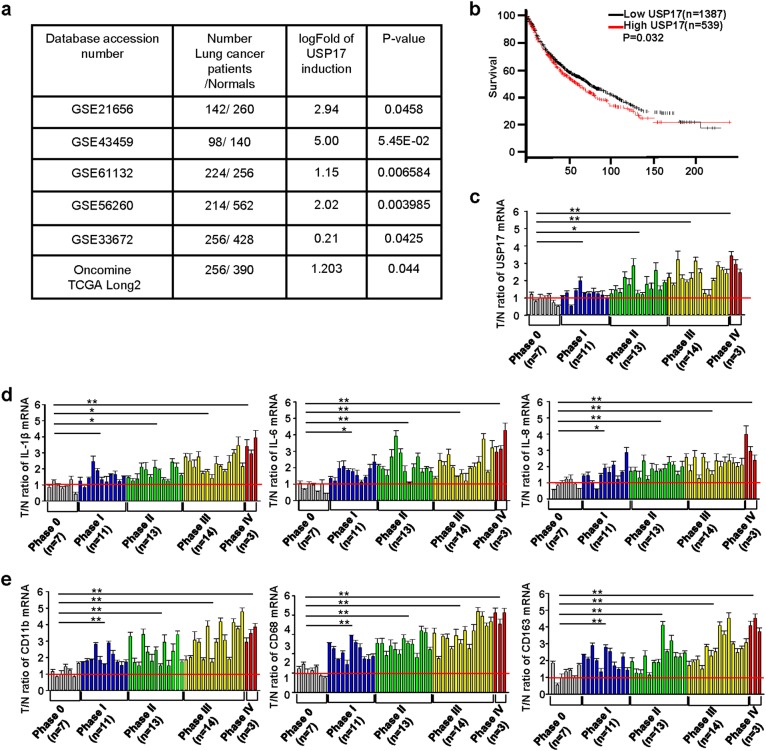
High expression of ubiquitin-specific peptidase 17, macrophages markers, and inflammatory mediators in lung cancers. **a** Different GEO and Oncomine datasets as indicated were analyzed for the induction of ubiquitin-specific peptidase 17 (USP17) in tissue samples obtained from patients with lung cancer. **b** Kaplan–Meier plotter analysis of USP17 expression and survival of patients with lung cancer. Correlation between USP17 expression and survival of patients with lung cancer was analyzed online by the Kaplan–Meier Plotter software. The data of patients with lung cancer in the database of KM plotter were collected from the GEO and Oncomine databases. **c**, **d** A set of cDNA array prepared from 48 normal or lung cancer tissues was subjected to RT-qPCR for analyzing the expressions of USP17 (**c**), inflammatory markers (**d**), and macrophage markers (**e**) as indicated. Clinic data of each sample are shown in Supplementary Table 1. Data represent mean ± standard deviation of three analysis, *P < 0.05; **P < 0.01

The tumor microenvironment contains abundant cytokines. Moreover, cancer cells interact with stromal cells such as macrophages to support tumor development [3–6]. Therefore, we hypothesized that the cytokine-inducible deubiquitinase USP17 should be highly expressed in cancer cells and function in modulating tumor growth. We analyzed its expression in normal tissues and lung cancer samples in six datasets from the GEO and Oncomine databases. The data revealed significantly higher USP17 expression in lung cancer samples than in normal tissues (Fig. 1a). Survival analysis of lung cancer data in KM plotter using an online Kaplan–Meier Plotter software revealed that patients with lung cancer exhibiting a high USP17 expression as high as 38.86% have a significantly lower survival rate than that of patients with a lower USP17 expression (Fig. 1b). To elucidate the mechanism of USP17 induction in lung cancers, we examined the expression of USP17 and macrophage and inflammatory markers in a set of cDNA array using 48 cDNA samples from patients with lung cancer with clinical data as shown in Supplementary Table 1. Consistent with the results obtained from the database analysis, lung cancer tissues showed a higher expression of USP17 than that in normal tissues. In addition, the expression level was elevated in parallel with the increase of lung cancer stages (Fig. 1c). In association with elevated USP17 expression, the levels of inflammatory mediators, including IL-1β, IL-6, and IL-8 were increased in lung cancer samples (Fig. 1d). The expression levels of macrophage markers, including cluster of differentiation (CD)11b, CD68, and CD163, were also increased (Fig. 1e). Moreover, correlations were observed between the increase in USP17 expression and the expression of inflammatory and macrophage markers in these lung cancer samples (Supplementary Figure 1a, b), suggesting a relationship among macrophage accumulation, inflammation, USP17 expression, and poor prognosis in lung cancers.

### Induction of USP17 expression in cancer cells by macrophages

**Fig. 2:**
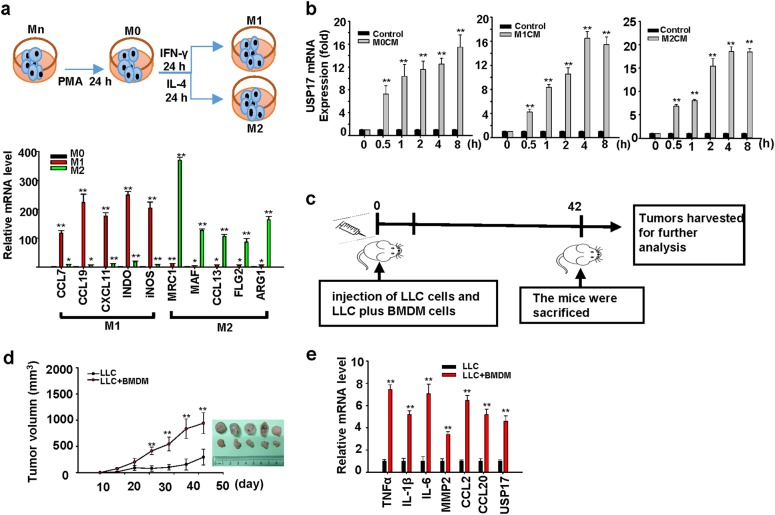
Role of macrophages in inducing ubiquitin-specific peptidase 17 expression in lung cancers. **a** Top panel: Schematic diagram for Mn THP-1 cells activation into M0 macrophages, and their polarization into M1 and M2 macrophages. Bottom panel: Polarization of M1 and M2 macrophages was characterized by real-time quantitative polymerase chain reaction (RT-qPCR) of their markers. **b** Induction of ubiquitin-specific peptidase 17 (USP17) in H1299 lung cancer cells by conditioned media from different macrophage types. USP17 expression was analyzed by RT-qPCR. **c–e** Mice were subcutaneously injected with 1 × 10^5^ of Lewis lung cancer (LLC) cells alone or a mixture of LLC cells and bone marrow-derived macrophages with a 7:3 ratio of a total number of 1 × 10^5^ into C57BL/6J mice following the schedule illustrated (**c**). Tumor volume was measured at the indicated time-points, and the mean tumor size was plotted (mean ± standard deviation, *n* = 5). The mice were killed after 42 days, tumors were collected and imaged (**d**). USP17 and inflammation-associated gene expressions in the collected tumors were analyzed by real-time quantitative PCR (**e**). Bars, data represent mean ± standard deviation of three independent experiments (**a**, **b**) or analysis (**e**), *P < 0.05; **P < 0.01 compared with the group of M0 macrophages (**a**), the group of control medium treatment (**b**), or the group of injection with LLC cells alone (**e**)

Macrophages are a large population of stromal cells and a key source of cytokines in the tumor microenvironment; thus, we investigated whether USP17 expression in cancer cells is induced by their interaction with macrophages. THP-1 monocytic cells (Mn) were activated by phorbol-12-myristate-13-acetate into M0 macrophages, which were further polarized with interferon-γ and IL-4 into M1 and M2 macrophages, respectively (Fig. 2a, top panel). Macrophage polarization was confirmed by real-time quantitative PCR of markers, such as CCL7, CCL19, CXCL11, INDO, and iNOS for M1 macrophages, and MCR1, MAF, CCL13, FLG2, and ARG1 for M2 macrophages [29, 30] (Fig. 2a, bottom panel). Conditioned media from these different macrophages were collected for culturing human H1299 lung cancer cells. Real-time quantitative PCR revealed that conditioned media from M0, M1, and M2 macrophages induced USP17 expression in lung cancer cells (Fig. 2b). The induction of USP17 expression in cancers by macrophages was further verified using a cancer animal model for studying the effects of macrophages on tumor growth. C57BL/6J mice were subcutaneously (SC) injected with 1 × 10^5^ Lewis lung cancer (LLC) cells or a 7:3 ratio mixture of 1 × 10^5^ LLC cells plus bone marrow-derived macrophages derived from C57BL/6J mice, as illustrated in Fig. 2c. Co-injection of macrophages resulted in in faster tumor growth compared with the injection of cancer cells alone, even when only 70% of cancer cells were coinjected (Fig. 2d), and also resulted in the upregulation of USP17 and inflammation-associated genes in tumors (Fig. 2e). These resemble the correlation among high expressions of macrophage markers, USP17, and inflammatory mediators observed in lung cancer samples (Fig. 1c–e). In addition, these results suggested that macrophages induced the expression of USP17 in tumors.

USP17 could be induced by cytokines secreted from macrophages because various cytokines, including TNF-α, IL-1β, IL-4, IL-6, IL-8, IL-10, CXCL12, CCL18, and CCL22, were able to induce USP17 expression in H1299 and D121 lung cancer cells (Supplementary Figure 2a, b). Moreover, USP17 promoter region analysis revealed different transcription factor binding sites, including those for transcription factors HIF-1, STAT3, STAT6, and NF-κB (Supplementary Figure 3), which are known to be activated on stimulating cancer cells by cytokines in a cross talk between TAMs and cancer cells [10, 14].

### USP17 promotes intrinsic inflammation and stimuli-activated inflammatory responses in lung cancer cells

**Fig. 3:**
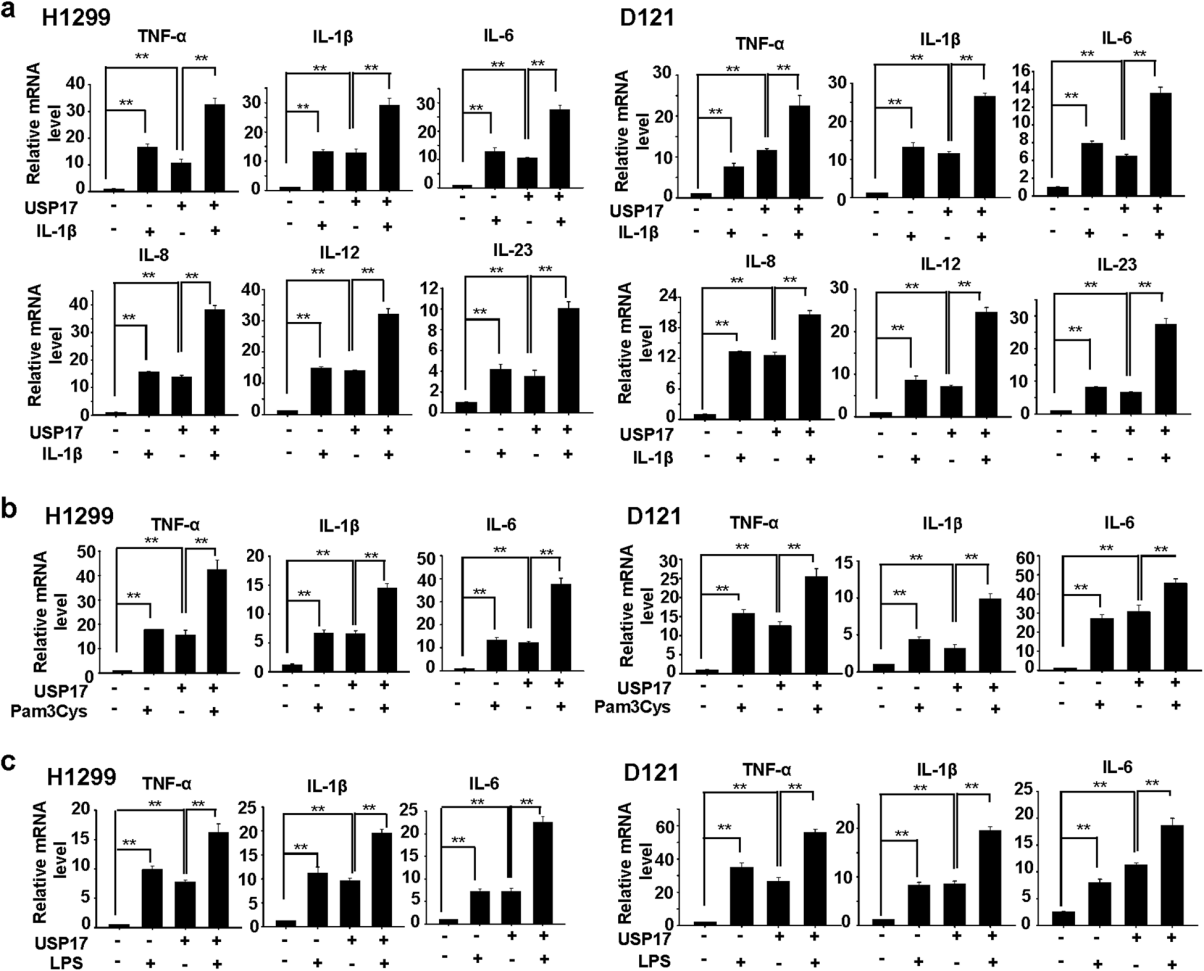
Role of ubiquitin-specific peptidase 17 in controlling intrinsic and stimuli-induced inflammatory responses in lung cancer cells. **a**–**c** H1299 and D121 lung cancer cells stably overexpressing ubiquitin-specific peptidase 17 or stably transfected with control vector were treated with or without 10 ng/ml interleukin-1β (**a**), 0.2 μg/ml Pam3cys (**b**), and 0.2 μg/ml LPS (**c**). The induction of cytokines in these cells was analyzed with real-time quantitative PCR. Results are shown as mean ± standard deviation of three independent experiments, **P < 0.01 between the indicated groups

We further investigated USP17 function in controlling inflammatory responses in lung cancer cells. Lung cancer H1299 and D121 cells stably overexpressing USP17 (expression levels shown in Supplementary Figure 4a, b) were treated with or without IL-1β. The production of various inflammatory cytokines, including TNF-α, IL-1β, IL-6, IL-8, IL-12, and IL-23, was measured by real-time quantitative PCR. USP17 overexpression in these cells enhanced basal production of these cytokines. IL-1β-induced cytokine production was further enhanced by USP17 overexpression in these cells (Fig. 3a). The effect of USP17 could be through the regulation of NF-κB activation since that was increased in parallel with the cytokine production in H1299 cells (Supplementary Figure 5a). In addition, in these cells, the expression of USP17 was induced by the IL-1β stimulation (Supplementary Figure 5b and Supplementary Figure 2a), which may provide some explanation for the additive but not the synergistic effect of USP17 overexpression plus IL-1β treatment in cytokine inductions. TLR ligands are potent inflammatory stimulants of cancer cells [13–15]. Thus, we investigated the role of USP17 in regulating TLR ligand-induced inflammatory responses. Cells were treated with Pam3Cys (TLR2 ligand) and LPS (TLR4 ligand) to induce inflammatory responses. Similar to its effect on IL-1β-induced inflammatory responses (Fig. 3a), USP17 enhanced cytokine production induced by TLR2 and TLR4 activation (Fig. 3b, c).

### USP17 promotes stemness and transformation ability of cancer cells

**Fig. 4:**
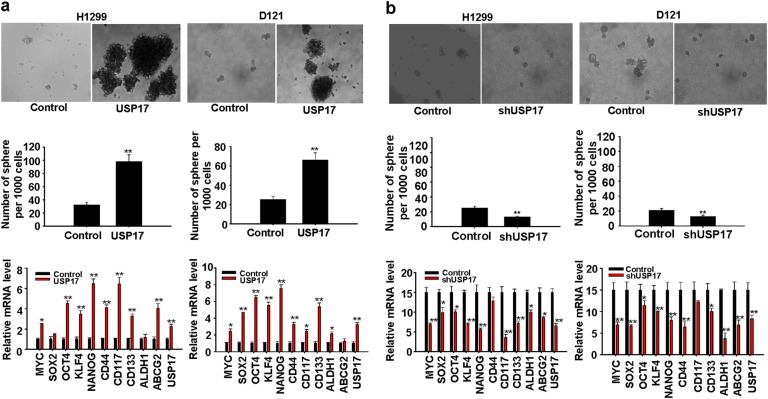
Role of ubiquitin-specific peptidase 17 in promoting stemness in lung cancer cells. **a**, **b** Control and ubiquitin-specific peptidase 17 (USP17) stably overexpressing (**a**), and control and USP17 short-hairpin RNA stably knockdown (**b**) H1299 and D121 lung cancer cells were grown in defined serum-free medium for sphere formation. Top panels: Cellular morphology of stemness-enriched spheres was monitored. Middle panels: The number of spheres in the medium was counted. Bottom panels: USP17 and stemness-associated gene expressions in cells were analyzed by real-time quantitative PCR. Data represent mean ± standard deviation of three independent experiments, *P < 0.05; **P < 0.01 compared with the control group

Inflammation and stemness are suggested to enhance each other and form a positive-feedback loop that promotes tumor growth [31, 32]. In addition, TAMs have been shown to enhance stemness in cancer cells [30]. Thus, we investigated USP17 function in regulating the stemness and transformation ability of lung cancer cells. We examined the sphere-forming ability of H1299 and D121 lung cancer cells stably overexpressing USP17 (expression levels are shown in Supplementary Figure 4a, b). USP17 expression promoted sphere formation (Fig. 4a, top and middle panels). Analysis of stemness-associated gene expressions using real-time quantitative PCR revealed elevated MYC, SOX2, OCT4, KLF4, NANOG, CD44, CD117, CD133, ALDH1, and ABCG2 expressions in USP17-overexpressing cells relative to those in control cells (Fig. 4a, bottom panels). In addition, using flow cytometric analysis, we verified that the number of D121 cells with detectable levels of CD117 and CD133 on the cell surface was increased in the USP17-overexpressed cells (Supplementary Figure 6). Conversely, stable USP17 knockdown in cancer cells using a short-hairpin RNA reduced their sphere-forming ability (Fig. 4b, top and middle panels, and knockdown levels are shown in Supplementary Figure 4a, b). Correlated with reduced USP17 expression, stemness-associated gene expressions were also reduced in these cells (Fig. 4b, bottom panels). We assessed the effect of USP17 expression on the transformation ability of cancer cells using cell proliferation and anchorage-independent growth assays. USP17 overexpression in H1299 and D121 lung cancer cells increased their proliferation (Supplementary Figure 7a). Moreover, in the anchorage-independent growth assay, significantly higher colony numbers were evident with USP17-overexpressing cells than with control cells (Supplementary Figure 7b). Thus, USP17 regulates stemness-associated properties and transformation ability of lung cancer cells.

### USP17 expression in cancer cells promotes macrophage recruitment and cytokine production by macrophages

**Fig. 5:**
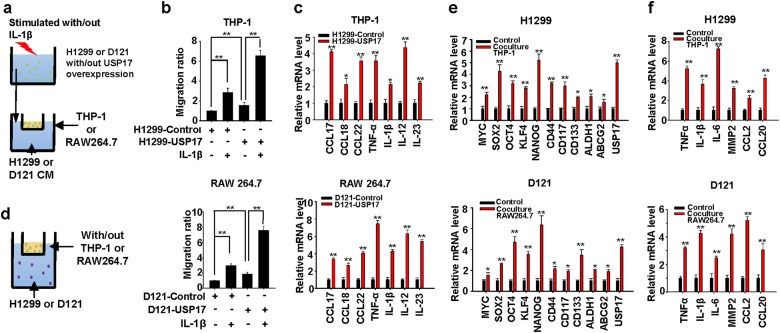
Role of ubiquitin-specific peptidase 17 in the interaction between macrophages and lung cancer cells to enhance inflammation and stemness in cancer cells. **a** Schematic diagram of the macrophage recruitment assay. Conditioned medium collected from control and ubiquitin-specific peptidase 17 (USP17)-overexpressing cancer cells stimulated with or without 10 ng/ml interleukin-1β were added to the lower chamber of 0.4-μm transwell plates. Macrophages were plated on the upper chamber and incubated at 37 °C for 8 h. **b** Macrophages that migrated into the lower chamber were fixed, stained with 0.05% crystal violet, and counted. **c** Macrophages were cultured with conditioned media from control and USP17-overexpressing cells at 37 °C for 8 h. Inflammatory gene expressions in macrophages were analyzed with real-time quantitative polymerase chain reaction (RT-qPCR). **d** Schematic diagram of coculturing macrophages and cancer cells. The upper chamber of 0.4-μm transwell plates was cultured with or without macrophages; cancer cells were plated on the lower chamber. **e**, **f** The cells were incubated at 37 °C for 8 h. Stemness-associated (**e**) and inflammatory (**f**) gene expressions in the cancer cells were analyzed using RT-qPCR. Data represent mean ± standard deviation of three independent experiments, *P < 0.05; **P < 0.01 between the indicated groups, or compared with the control group

Because USP17 overexpression in cancer cells promotes intrinsic inflammation and stimuli-induced inflammatory responses (Fig. 3), which can result in an inflammatory tumor microenvironment favorable for macrophage recruitment, we further investigated the role of USP17 in cancer cells for their interaction with macrophages. A macrophage recruitment assay was performed (Fig. 5a). Control and USP17-overexpressing H1299 and D121 lung cancer cells were treated with or without IL-1β. Conditioned media collected from these cells were analyzed for their ability to promote macrophage migration. USP17 overexpression in cancer cells promoted macrophage recruitment (Fig. 5b) and induced cytokine production by macrophages (Fig. 5c). Macrophages promoted USP17 expression in cancer cells (Fig. 2), therefore we turned to examine the role of macrophages in promoting inflammation and stemness in cancer cells. Macrophages and cancer cells were cocultured in two-chamber transwell plates (Fig. 5d). Gene induction was analyzed by real-time quantitative PCR. Correlated with upregulated USP17 expression (Fig. 5e), stemness-associated and inflammation-associated gene expressions were upregulated when cancer cells interacted with macrophages (Fig. 5e, f), suggesting a role of USP17 in the interaction between macrophages to increase inflammation and stemness in cancer cells.

### USP17 contains binding motifs that allow it to interact with and disrupt the protein-degradation ability of the TRAF2/TRAF3 complex

**Fig. 6:**
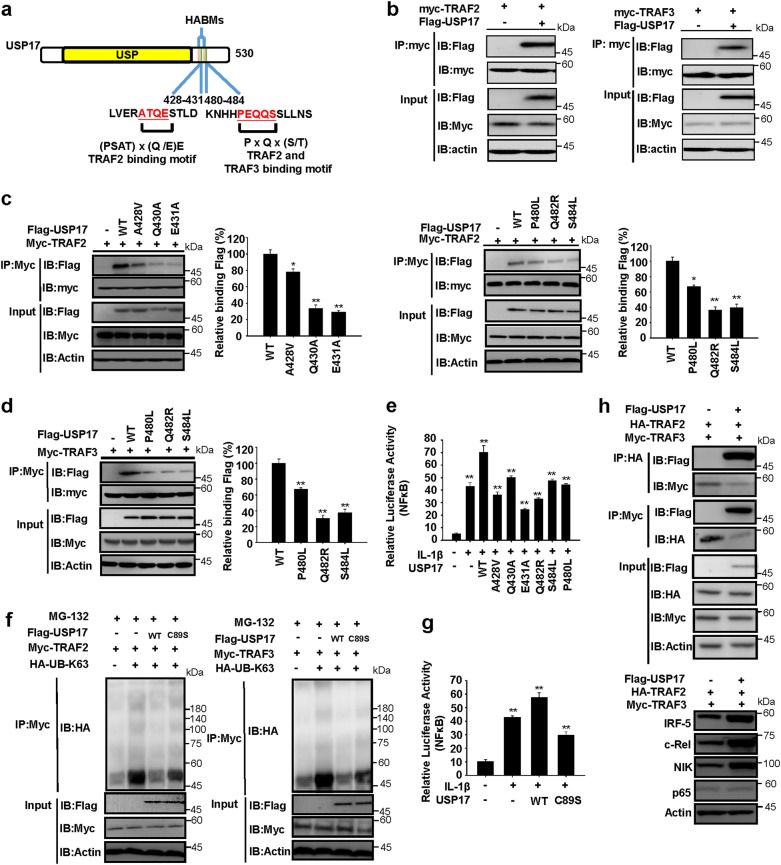
Ubiquitin-specific peptidase 17 disrupts formation of the TNFR-associated factor 2/TNFR-associated factor 3 complex and stabilizes their client proteins. **a** TNFR-associated factor (TRAF) 2-binding and TRAF3-binding motifs were identified in ubiquitin-specific peptidase 17 (USP17) as shown in the schematic diagram. USP Ubiquitin-specific protease, HAMBs hyaluronan-binding motifs. Numbers show the amino-acid residue positions. **b** USP17 was coexpressed with TRAF2 (left panel) or TRAF3 (right panel) in HEK293 cells, and their interaction was analyzed by immunoprecipitation and immunoblotting with the antibodies indicated. **c** Different amino-acid residues in the two TRAF2-binding motifs of USP17 were mutated. Wild-type (wt) USP17 and USP17 mutants were coexpressed with TRAF2 in HEK293 cells, and the requirement of the amino-acid residues in the binding motif of USP17 for TRAF2 binding was analyzed by immunoprecipitation and immunoblotting with the antibodies indicated. **d** Different amino-acid residues in the TRAF3-binding motifs of USP17 were mutated. Wt USP17 and these mutants were coexpressed with TRAF3 in HEK293 cells, and the requirement of the amino-acid residues in the binding motif of USP17 for TRAF3 binding was analyzed by immunoprecipitation and immunoblotting with the antibodies indicated. **e** HEK293 cells were co-transfected with a nuclear factor-κB (NF-κB)-controlled luciferase-reporter plasmid and expression vectors encoding wt USP17 and different USP17 mutants. These cells were treated with or without interleukin (IL)-1β (10 ng/ml) and the relative luciferase activities were analyzed to determine the requirement of TRAF2 and TRAF3 binding for regulating NF-κB activation by USP17. **f** HEK293 cells were co-transfected with expression vectors for wt USP17, C89S USP17, UB-K63, TRAF2 (left panel), or TRAF3 (right panel) and treated with MG132. The effects of wt USP17 and C89S USP17 on the K63-linked ubiquitination of TRAF2 and TRAF3 were analyzed by immunoprecipitation and immunoblotting with the antibodies indicated. **g** HEK293 cells were co-transfected with an NF-κB-controlled luciferase-reporter plasmid and expression vectors encoding wt USP17 and C89S USP17. These cells were treated with or without IL-1β (10 ng/ml) and the relative luciferase activities were analyzed to determine the requirement of deubiquitinase activity for regulating NF-κB activation by USP17. **h** HEK293 cells were co-transfected with expression vectors for USP17, TRAF2, and TRAF3, and the effects of USP17 on blocking TRAF2/TRAF3 complex formation (top panel) and stabilizing the client proteins of this complex (bottom panel) were analyzed by immunoprecipitation and immunoblotting with the antibodies indicated. Each set of blots is representative of three independent experiments. In the bar figures, the data represent mean ± standard deviation of three independent experiments, *P < 0.05; **P < 0.01 compared with the wt USP17 group (**c**, **d**), or the group without IL-1β treatment (**e**, **g**)

We assessed structural and functional mechanisms by which USP17 enhanced inflammatory responses and analyzed USP17 protein sequence. Besides the USP domain and hyaluronan-binding motifs (HABMs) [27, 28]. We identified two TRAF2-binding motifs in the USP17 protein: first located at position 428–431 and second at position 480–484. The second motif was also a TRAF3-binding motif (Fig. 6a) [33, 34]. We showed that interactions occur between USP17 and TRAF2 and TRAF3 by their co-immunoprecipitation when coexpressed in HEK293 cells (Fig. 6b). Similarly, coimmunoprecipitations of endogenous USP17 and TRAF2 and TRAF3, respectively, were observed in LLC cells (Supplementary Figure 8a). Furthermore, we demonstrated the requirement of the identified motifs for these interactions; mutation of residues in the first binding motif in USP17 reduced its ability to coimmunoprecipitate with TRAF2, and mutation of the second motif reduced its ability to coimmunoprecipitate with both of TRAF2 and TRAF3 (Fig. 6c, d). We investigated the requirement of TRAF2 and TRAF3 binding for enhancing inflammatory responses by USP17 in IL-1β-stimulated HEK293 cells and LLC cells using an NF-κB-driven luciferase-reporter assay. USP17 mutants with less able to bind TRAF2 and TRAF3 were less able to promote NF-κB activation (Fig. 6e and Supplementary Figure 8b). TRAF2 and TRAF3 are known to undergo K63-linked self-ubiquitination for protein–protein interactions [25, 26], therefore we also examined whether USP17 promotes TRAF2 and TRAF3 deubiquitination, and whether this deubiquitination activity is required for promoting inflammatory responses by USP17. It is known that cysteine residue 89 in the USP domain is required for the deubiquitination activity of USP17 [35–37], thus this cysteine was mutated into a serine residue (C89S) to generate an inactive mutant. When coexpressed in HEK293 cells, wild-type USP17 promoted K63-linked TRAF2 and TRAF3 deubiquitination, whereas the C89S mutant did not (Fig. 6f). In addition, the C89S mutant could not enhance IL-1β-induced NF-κB activation, in contrast to wild-type USP17 in HEK293 cells and LLC cells (Fig. 6g and Supplementary Figure 8c). We investigated the role of USP17 in controlling TRAF2/TRAF3 complex formation. USP17 overexpression blocked TRAF2 and TRAF3 binding (Fig. 6h, top panel) and increased the stability of client proteins of the TRAF2/TRAF3 complex, including NIK, c-Rel, and IRF5 (Fig. 6h, bottom panel). Therefore, the function of USP17 in enhancing inflammation and stemness in cancer cells may result from its ability to bind and disrupt the TRAF2/TRAF3 complex.

### USP17 drives a positive-feedback interaction between macrophages and cancer cells to promote tumor growth

**Fig. 7:**
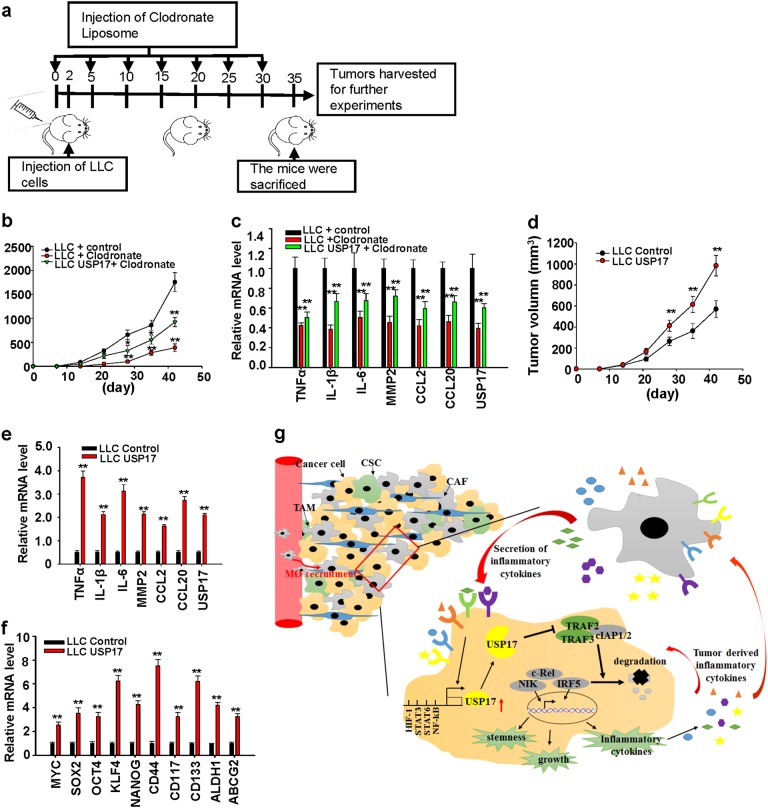
Role of ubiquitin-specific peptidase 17 in the positive-feedback loop of the interaction between macrophages and lung cancer cells to promote tumor growth. **a** To investigate the effect of macrophages on the induction of ubiquitin-specific peptidase 17 (USP17) expression and tumor growth, C57BL/6 mice with or without injection of clodronate liposomes for macrophage depletion were subcutaneously inoculated with 5 × 10^5^ of LLC cells stably transfected with control vector or LLC cells stably overexpressing USP17 following the illustrated schedule. **b** Tumor growth rates were monitored. **c** These mice were killed on day 42, USP17 and inflammatory gene expressions in tumors were analyzed by real-time quantitative PCR (RT-qPCR). **d**–**f** To investigate the effect of USP17 expression in tumor cells on tumor growth, C57BL/6 mice were inoculated with 1 × 10^5^ control or USP17-overexpressing LLC cells and tumor growth rates were monitored (**d**). These mice were killed on day 42. USP17 and inflammatory gene expressions in tumors were analyzed by RT-qPCR (**e**). Stemness-associated gene expressions in tumors were analyzed by RT-qPCR (**f**). Bars, data represent mean ± standard deviation of three independent analysis, **P < 0.01 compared with LLC and control (**b**, **d**), and compared with the control group (**c**, **e**, **f**). **g** Illustration of the role of ubiquitin-specific peptidase 17 in the positive-feedback loop of the interaction between macrophages and lung cancer cells to promote tumor growth. In lung cancers, macrophages induce ubiquitin-specific peptidase 17 (USP17) expression in cancer cells. USP17 stabilizes and enhances NIK-, c-Rel-, and IRF5-mediated inflammation-associated and stemness-associated gene expressions by disrupting the TNFR-associated factor (TRAF) 2/TRAF3 complex. These effects further recruit macrophages into tumors, driving a positive-feedback interaction between macrophages and cancer cells to promote progression and malignancy of lung cancers

We further investigated the role of USP17 in control of positive interaction between macrophages and cancer cells with animal models of cancer. Injection of clodronate-containing liposomes into C57BL/6J mouse was able to deplete about 70% of the mouse macrophages (Supplementary Figure 9). Control and USP17-overexpressing LLC cells (5 × 10^5^) were injected into C57L/B6 mice treated with or without clodronate-containing liposomes to deplete macrophages as illustrated in Fig. 7a. In contrast to the results shown in Fig. 2c–e that macrophages promote expression of USP17 and inflammation-associated gene in tumors and accelerate tumor growth, depletion of macrophages in host animals reduced tumor growth, USP17 expression, and inflammatory cytokine productions (Fig. 7b, c). In addition, these effects resulted by macrophage deletion were partially restored by stable USP17 overexpression in cancer cells (Fig. 7b, c). We further examined the effect of USP17 expression on tumor growth by sc injection of 1 **×** 10^5^ control or LLC cells stably overexpressing USP17 into C57L/B6 mice. USP17 overexpression in cancer cells promoted tumor growth (Fig. 7d) and increased inflammation-associated and stemness-associated gene expressions in tumors (Fig. 7e, f). Thus, these results demonstrate the positive role of USP17 in regulating the interaction between macrophages and cancer cells to enhance inflammation and stemness in tumors for promoting tumor growth (Fig. 7g).

## Discussion

Inflammation contributes to tumorigenesis and lung cancer development. Macrophages are inflammatory cells that accumulate in the tumor microenvironment throughout tumor progression [3–5]. Thus, the interaction between macrophages and cancer cells and the subsequent responses could be a primary cause of malignancy. In this study, we found that high USP17 expression was associated with increased inflammatory and macrophage marker expressions in lung cancers. These findings suggest a connection among USP17 expression, macrophage accumulation, and inflammation in lung cancer. In addition, our result of database analysis revealed a correlation between high USP17 expression and poor prognosis in lung cancers. In this regard, this result is consistent with previously reported that patients with USP17 positive tumors had significantly reduced survival than patients with USP17 negative tumors, and knockdown of USP17 inhibited tumorigenesis and growth of non-small-cell lung cancer in xenograft animal model [38, 39], although inhibitory effect of USP17 on cancer cell growth was also reported, for examples co-treatment of bromodomain and extra-C terminal domain protein inhibitors and histone deacetylase inhibitors has been shown to induce expression of USP17 and reduces breast cancer cell viability [40]. USP17 and its substrate SDS3 are involved in the inhibition of anchorage-independent tumor cell growth [41].

USP17 was reported to reverse K63-linked ubiquitination of Ras-converting enzyme 1 and reduced its activity for post-translational modification of Ras GTPases [42]. Another study identified high USP17 expression in lung, colon, esophageal, and cervical tumors and showed that USP17 expression is necessary for cell cycle progression by regulating GTPases [43]. The K63-linked deubiquitinase activity of USP17 is also involved in regulating SDS3, reducing histone deacetylase activity in cancer cells [35]. Conversely, USP17-mediated K48-linked deubiquitination protects Cdc25A from proteasomal degradation and promoted oncogenic transformation [36]. USP17 also modulates both K48-linked and K63-linked ubiquitination of RIG-I and regulates immune responses of IL-33 [37, 44]. In this study, we showed that USP17 reduced K63-linked ubiquitination of TRAF2 and TRAF3. Therefore, USP17 can remove both K48-linked and K63-linked ubiquitination of target proteins to regulate their cellular functions.

Besides the HABMs and USP domain [27, 28], we identified binding motifs for TRAF2 and TRAF3 in USP17. TRAF2 and TRAF3 form a complex involving cIAPs that possessesE3 ligase activity and performs K48-linked ubiquitination on target proteins, including NIK, c-Rel, and IRF5 for proteolytic degradation. These target proteins are involved in controlling inflammatory cytokine expressions [25, 45–47], thus proteins interacting with TRAF2 or TRAF3 may disrupt the TRAF2/TRAF3 complex to increase inflammatory responses. Consistently, USP17 expression in lung cancer cells enhanced basal and stimuli-induced inflammatory responses by stabilizing NIK, c-Rel, and IRF5 through disrupting the TRAF2/TRAF3 complex.

In this study, we found that USP17 expression in lung cancer cells increases inflammation-associated and stemness-associated gene expressions. Previous studies have shown that NF-κB-mediated inflammatory responses promote CSC phenotype by directly inducing NF-κB-controlled stemness-associated gene expressions and indirectly activating these genes through cytokines generated in inflammatory responses [16–18]. Two recent studies demonstrated that a direct interaction between USP17 and Snail1, a key transcription factor of epithelial–mesenchymal transition (EMT) regulates EMT phenotype and cancer invasion. USP17 stabilizes Snail1 through its deubiquitination activity, and USP17 knockdown or inhibition can promote Snail1 degradation and suppresses cancer invasion and metastasis [48, 49]. The EMT phenotype can initiate the metastasis of cancer cells and their dedifferentiation into CSCs [18–20]. Hence, USP17 may promote malignancy by directly controlling NF-κB-mediated inflammation, and directly and indirectly controlling stemness.

USP17 is a DUB enzyme inducible by various cytokines [27, 28, 49, 50]. Lung cancers exhibit high USP17 expression because tumor cells occupy a microenvironment containing abundant cytokines and other mediators that support tumor growth. Macrophages constitute a major cytokine-producing population in tumors. TAMs are not a single uniform population but exhibit features of a spectrum of states with M1 (or classically activated) and M2 (or alternatively activated) phenotypes at opposite ends. A simplistic model of their function is that M1 TAMs perform proimmunogenic functions that favor the immunosurveillance of malignant cells, whereas M2 TAMs have immunosuppressive effects and perform protumor functions. Nevertheless, macrophage accumulation in tumors is often associated with poor prognosis [7, 8, 51, 52]. Thus, the role of TAMs in tumor progression may not be limited to their effect on immunosurveillance, and their alternative effects on cancer cells should be considered.

As illustrated in Fig. 7g, USP17 expression in lung cancer cells was induced by cytokines secreted by macrophages with either M1 or M2 properties. Increased USP17 expression disrupted the TRAF2/TRAF3 complex formation and stabilized its target proteins, leading to elevated inflammation, stemness, and transformation ability in lung cancer cells and increased macrophage recruitment into tumors. These findings suggest a novel mechanism for the protumor effect of tumor-associated macrophages through induction of USP17 in lung cancer cells, and high USP17 expression contributes to a positive-feedback interaction between macrophages and cancer cells that drives lung cancer progression. High USP17 expression in lung cancers also implies that this protein represents diagnostic marker. Inhibitors for USPs are currently under development suggesting that these deubiquitinases are potential therapeutic targets [53, 54]. Moreover, a small molecular-weight inhibitor, WP1130,was recently shown to inhibit USP17 function and cancer metastasis [49]. Taken together, our findings, together with the development of USP17 inhibitors, suggesting that USP17 could be a therapeutic target for lung cancer treatment.

## Materials and methods

### Bioinformatics analysis

The Oncomine (https://www.oncomine.org/) and GEO databases (https://www.ncbi.nlm.nih.gov/gds/) were searched to analyze the expression profiles of USP17 and other genes in normal and tumor tissues. The KM plotter (http://kmplot.com/analysis/) [55, 56] was analyzed online for the survival Kaplan–Meier estimates of patients with different USP17 expression levels. Transcription factor binding sites in the promoter region were analyzed with software available at the Gene Promoter Miner website (http://gpminer.mbc.nctu.edu.tw/).

### Cell lines and cell culture

Human H1299 lung cancer, human embryonic kidney (HEK) 293, mouse D121, LLC lung cancer, and RAW264.7 cells were grown in Dulbecco’s modified Eagle’s medium (DMEM) supplemented with 10% fetal bovine serum (FBS). Human THP-1 monocytic cells and mouse 4T1 breast cancer cells were grown in Roswell Park Memorial Institute 1640 medium supplemented with 10% FBS. The cells were cultured at 37 °C in an atmosphere containing 5% CO_2_. The D121 lung cancer cell line was developed by Dr. L. Eisenbach (Weizman Institute, Rehovot, Israel [57]). All of the other cell lines are available from American Type Culture Collection. These cells were periodically cultured with 25 μg/ml of Plasmocin (Invivogen, San Diego, USA) to prevent mycoplasma contamination and periodically checked with an EZ-PCR Mycoplasma test (Biological Industries, Kibbutz Beit Haemek, Israel) for contamination.

### Lentiviral expression vector construction, infection, and stable cell lines

To generate a USP17 lentiviral expression vector, USP17 cDNA was cloned into the *Nhe*I/*Eco*RI sites of the pLAS5w vector (RNAi Core of Academia Sinica, Taiwan) for protein expression. The lentivirus was produced by harvesting culture supernatants obtained upon transfecting 293T cells with the generated constructs and packaging plasmids using TransIT-LT1 (Mirus Bio LLC, Madison, WI, USA). Cancer cell lines were spin infected by plating cells in 12-well plates in the presence of lentiviral supernatants and 8 μg/ml polybrene (Sigma-Aldrich Corp.), followed by centrifugation at 1100×*g* for 30 min. The cells were subjected to selection with puromycin (3 ng/ml) to obtain stable cell lines.

### Reverse-transcription and real-time quantitative PCR analyses

Total RNA was purified from cells using TRIzol (Invitrogen, Carlsbad, CA, USA) according to the manufacturer’s protocol. Reverse-transcription was performed using the Super Script III first-strand synthesis system (Invitrogen) and oligo-dT primers for first-strand cDNA synthesis. Quantitative PCR was performed with gene-specific primers (Supplementary Table 2) using an ABI PRISM 7900*HT* sequence detection system (Applied Biosystems, Foster City, CA, USA) and KAPA SYBR Fast qPCR Kit (KK4605) for gene expression analysis. mRNA expression was normalized to that of β-actin.

### Ubiquitination assays

Expression vectors encoding tagged TRAF2 or TRAF3 were transfected into HEK293 cells with Flag-USP17 and HA-Ubiquitin. The cells were treated with 10 μM MG132 for 24 h and then lysed with Nonidet P-40 lysis buffer containing complete protease inhibitor cocktail. Ubiquitination of the indicated protein was analyzed by immunoprecipitation, followed by immunoblotting using the indicated antibodies.

### Polarization of macrophages

THP-1 (Mn cells) were differentiated with phorbol-12-myristate-13-acetate (100 ng/ml; EMD Calbiochem, La Jolla, CA, USA); the media were changed the next day and subsequently every 2 days for 6 days. Polarization of the resting differentiated macrophages (M0 cells) was performed by 24 h treatment with 20 ng/ml of interferon-γ (R&D Systems, Inc., Minneapolis, MN, USA) for M1-like polarization and 30 ng/ml of IL-4 (R&D Systems, Inc.) for M2-like polarization. The cells were washed and incubated with fresh medium. Conditioned media from these polarized cells was harvested after 24 h and frozen at −80 °C.

### Macrophage recruitment analysis

Conditioned media collected from control and cancer cells stably overexpressing USP17 were added to the lower chamber of transwell plates containing polyethylene terephthalate membrane inserts with a 5-μm pore size (Corning Life Sciences, Tewksbury, MA, USA). THP-1 or RAW264.7 cells were plated on the upper chamber and incubated for 8 h at 37 °C. Migratory cells were fixed, stained with 0.05% crystal violet, and counted in five randomly selected fields.

### Sphere-formation assay

For sphere formation, cells were cultured at a density of 1 × 10^3^ on ultra-low attachment six-well plates (Corning Life Sciences) in culture medium consisting of serum-free DMEM/F12-K medium, N2 supplement (Invitrogen), 20 ng/ml EGF, and 20 ng/ml bFGF. The spheres were then photographed and counted.

### Animal models of cancer

Animal experiments were approved by the Institutional Animal Care and Use Committee of the National Health Research Institutes, Miaoli, Taiwan. Male and female with age from 1–6 months of C57BL/6J mice were maintained and handled in accordance with the stated guidelines of 3Rs (replacement, reduction, and refinement) for the research design and statistical analysis of experiments using laboratory animals. Mice included in the study were randomized and blinded to the group assignment. Three different animal models were employed to investigate the in vivo effect of macrophages on the induction of USP17 and tumor growth. First, 1 × 10^5^ of mouse LLC cells, or a mixture of LLC cells and bone marrow-derived macrophages at a ratio of 7:3, were subcutaneously (sc) injected into C57BL/6J mice. Second, macrophages in C57BL/6 mice were depleted by injection of clodronate-containing liposomes (FormuMax Scientific Inc., Sunnyvale, CA, USA). An initiation dose of 200 μl of clodronate liposomes was injected intraperitoneally into C57BL/6J mice 2 days before the sc injection of 5 × 10^5^ of mouse lung cancer LLC cells. To prevent the repopulation of macrophages, the mice were repeatedly injected with 100 μl of clodronate liposomes every 5 days. Macrophage depletion was maintained throughout the experimental period. Third, to evaluate the effect of USP17 expression in lung cancer cells on tumor growth, 1 × 10^5^ of control cells and LLC cells stably overexpressing USP17 were sc injected into C57BL/6J mice. The mice were monitored for tumor growth and killed at the indicated times for different analyses. Tumor volume (TV) was calculated using the following formula:

$${\mathrm{TV}}\left( {{\mathrm{mm}}^3} \right) = \left( {{\mathrm{Length}} \times {\mathrm{width}}^2} \right)/2.$$

### Statistical analysis

Statistical analysis was performed on data derived from three or more independent experiments using the Student’s *t*-test. Comparison between two groups was performed using two-tailed *t*-test. Correlation between two groups was determined by analysis of Pearson’s correlation coefficient. All data are presented as means ± standard deviation. A *P*-value of <0.05 was considered to represent statistically significant differences between the experimental groups.

Reagents and antibodies, plasmid construction, transfection and luciferase-reporter analysis, immunoblotting and co-immunoprecipitation analysis, anchorage-independent growth, and cell proliferation assay are described in the Supplementary materials and methods.

## Electronic supplementary material

Supplementary Information
