# Correction: USP17 mediates macrophage-promoted inflammation and stemness in lung cancer cells by regulating TRAF2/TRAF3 complex formation

**DOI:** 10.1038/s41388-019-0831-5

**Published:** 2019-05-30

**Authors:** Chih-Hao Lu, Da-Wei Yeh, Chao-Yang Lai, Yi-Ling Liu, Li-Rung Huang, Alan Yueh-Luen Lee, S. -L. Catherine Jin, Tsung-Hsien Chuang

**Affiliations:** 1 Immunology Research Center, National Health Research Institutes, Miaoli, Taiwan; 2 Department of Life Sciences, National Central University, Zhongli District, Taoyuan City, Taiwan; 3 Institute of Molecular and Genomic Medicine, National Health Research Institutes, Miaoli, Taiwan; 4 National Institute of Cancer Research, National Health Research Institutes, Miaoli, Taiwan; 5 Program in Environmental and Occupational Medicine, Kaohsiung Medical University, Kaohsiung, Taiwan

**Keywords:** Cancer microenvironment, Immunosurveillance, Cancer stem cells

**Correction to: Oncogene** (2018) 37:6327–6340

10.1038/s41388-018-0411-0

Following the publication of this article, the authors have noticed errors in Fig. 1a, b that were caused due to combined and accidental placement of unrelated data obtained from database search. The authors have corrected these figures. The amended version of Fig. 1 is shown below. Accordingly, the fifth sentence in the first subsection of the Results section is corrected as “Survival analysis of lung cancer data in cBioPortal using an online Kaplan–Meier analysis software revealed that patients with lung cancer exhibiting the top 9.2% high USP17 expression have a significantly lower survival rate than that of patients with a lower USP17 expression (Fig. 1b).” This correction does not affect the conclusions depicted in Fig. 1a regarding high USP17 expression in lung cancers and those depicted in Fig. 1b for the correlation between high USP17 expression and lower survival rate of patients with lung cancer. These conclusions are also supported by other published data [1–3] and our experimental data shown in Fig. 1c, wherein the analysis of USP17 expression in the cDNA array of lung cancers by RT-qPCR demonstrated high expression of USP17 in lung cancers, in particular lung cancers at high stages. The high expression and the pro-tumoral effect of USP17 (DUB3/USP17L2) have also been reported for other tumor types [4–7]. The authors express their sincere apologies for these errors and any inconvenience caused.

**Fig. 1:**
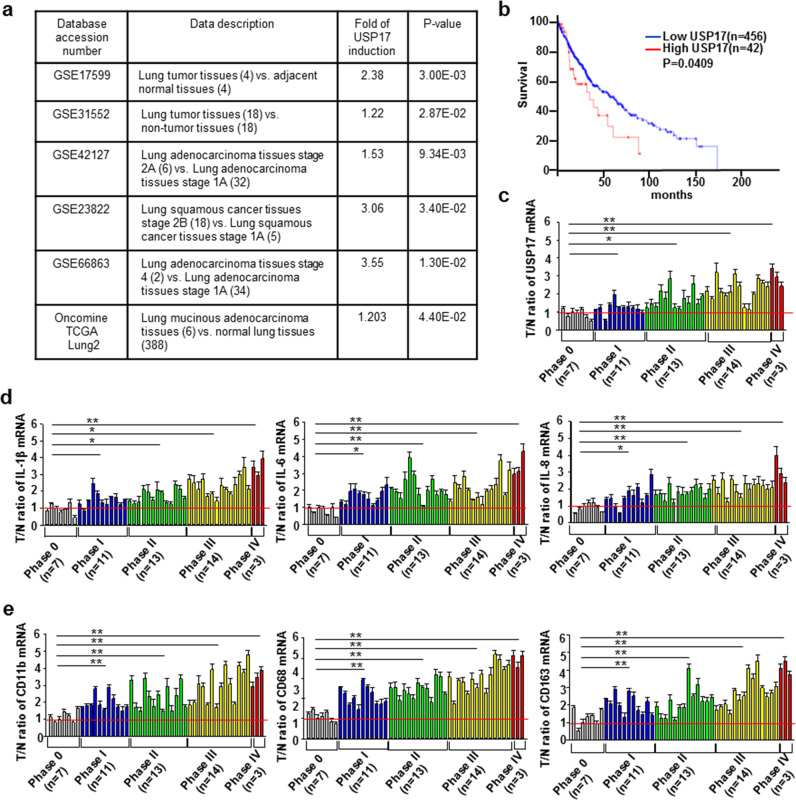
High expression of ubiquitin-specific peptidase 17, macrophages markers, and inflammatory mediators in lung cancers. **a** Different GEO and Oncomine datasets as indicated were analyzed for the induction of ubiquitin-specific peptidase 17 (USP17) in tissue samples obtained from patients with lung cancer. **b** Kaplan–Meier analysis of USP17 expression and survival of patients with lung cancer. Correlation between USP17 expression and survival of patients with lung cancer was analyzed online by the cBioPortal software. The data of patients with lung cancer in the database of cBioPortal were from the Cancer Genome Atlas (TCGA). **c**–**d** A set of cDNA array prepared from 48 normal or lung cancer tissues was subjected to RT-qPCR for analyzing the expressions of USP17 (**c**), inflammatory markers (**d**), and macrophage markers (**e**) as indicated. Clinic data of each sample are shown in Supplementary Table 1. Data represent mean ± standard deviation of three analysis, *P < 0.05; **P < 0.01
