# Supplementary material for: USP17 mediates macrophage-promoted inflammation and stemness in lung cancer cells by regulating TRAF2/TRAF3 complex formation
Source: Oncogene. 2018 Jul 23;37(49):6327–40. doi: 10.1038/s41388-018-0411-0 (PMC6283856; doi:10.1038/s41388-018-0411-0)

USP17 mediates macrophage-promoted inflammation and stemness in lung cancer cells by regulating TRAF2/TRAF3 complex formation

Chih-Hao Lu[1,2] • Da-Wei Yeh[1] • Chao-Yang Lai[1] • Yi-Ling Liu[1] • Li-Rung Huang[3] • Alan Yueh-Luen Lee[4] • S.-L. Catherine Jin[2] • Tsung-Hsien Chuang[1,5]

[1]Immunology Research Center, National Health Research Institutes, Miaoli, Taiwan
[2]Department of Life Sciences, National Central University, Zhongli District, Taoyuan City, Taiwan
[3]Institute of Molecular and Genomic Medicine, National Health Research Institutes, Miaoli, Taiwan
[4]National Institute of Cancer Research, National Health Research Institutes, Miaoli, Taiwan
[5]Program in Environmental and Occupational Medicine, Kaohsiung Medical University Kaohsiung, Taiwan

Tsung-Hsien Chuang
thchuang@nhri.org.tw

Supplementary Materials and Methods

Reagents and antibodies

Antibodies against human c-Rel, and mouse Cluster of differentiation (CD) 68 and CD206 were purchased from Abcam (Cambridge, UK). Anti-human IRF5 and NIK antibodies were purchased from Cell Signaling Technology, Inc. (Danvers, MA, USA). Anti-human USP17 antibodies were purchased from Abcam and OriGene (Rockville, MD, USA). Anti-mouse USP17 antibody was purchased from abnova (Taipei City, Taiwan). Anti-mouse TRAF2 and TRAF3 antibodies were purchased from Abcam. Anti-mouse CD117, CD133, CD145 and F4/80 antibodies were from BioLegend (San Diego, CA, USA). Recombinant human TNF-α, IFN-γ, IL-1β, IL-4, IL-6, IL-8, IL-10, CXCL12, CCL22, EGF, and FGF were purchased from Peprotech (Rocky Hill, NJ, USA). Anti-Flag antibody and chemical reagents were purchased from Sigma-Aldrich Corp. (St. Louis, MO, USA). Reagents for the luciferase assay were purchased from Promega Corp. (Madison, WI, USA). Lung cancer cDNA array was purchased from OriGene (Rockville, MD, USA).

Plasmid construction

Expression constructs for human USP17, TNFR-associated factor (TRAF) 2, and TRAF3 were generated through polymerase chain reaction (PCR) amplification of the corresponding protein-coding regions from a first-strand cDNA library derived from human spleen cells. Expression vectors for USP17 mutants and double mutants were generated by two-step PCR amplification. The amplified DNA fragments were cloned into the pRK5 vector for protein expression.

Transfection and luciferase-reporter analysis

For NF-κB activation assays, HEK293 cells were cotransfected with an NF-κB-driven luciferase-reporter plasmid and the indicated expression plasmids using polyethylenimine. The next day, the cells were treated with different stimuli for 7 h, followed by lysis of the cells and the determination of luciferase activity. Relative luciferase activities were calculated as fold inductions relative to unstimulated controls.

Immunoblotting and co-immunoprecipitation analysis

Protein extracts were prepared from whole cells. The cells were lysed with Nonidet P (NP)-40 lysis buffer (100 mM NaCl, 50 mM Tris-Cl pH 7.5, 0.5 mM ethylenediaminetetraacetic acid, 1% NP-40) containing complete protease inhibitor cocktail (Roche Life Science, Penzberg, Germany). The cell lysates were separated by sodium dodecyl sulfate-polyacrylamide gel

electrophoresis and transferred to polyvinyl difluoride membranes (Santa Cruz Biotechnology, Dallas, Texas, USA). The membranes were blocked for 1 h with 5% skimmed milk in TBST buffer (50 mM Tris-Cl pH 7.5, 150 mM NaCl, 0.1% Tween-20) and incubated for 1 h with the indicated primary antibodies in TBST containing 2% skimmed milk. The membranes were subsequently washed in TBST and incubated for 1 h with horseradish peroxidase (HRP)-conjugated secondary antibodies. The immune-reactive bands were visualized using a chemiluminescent HRP substrate and UVP BioSpectrum Imaging System. β-actin was used as a loading control. For immunoprecipitation (IP) and co-IP, the cell lysates were incubated with the indicated antibodies plus Protein A/G Magnetic Beads (Thermo Fisher Scientific, Waltham, MA, USA) at 4°C overnight to form immunocomplexes. After extensive washing with lysis buffer, the immunocomplexes were analyzed by immunoblotting.

Anchorage-independent growth

Bottom agar–medium mixture (DMEM, 10% FBS, 0.8% agarose) was added to each well of six-well cell culture plates and allowed to solidify. Top agar–medium mixture (DMEM, 10% FBS, 0.4% agarose) containing cells was added, followed by the addition of growth medium, and incubated at 37°C. To photograph and count the colonies, the plates were fixed with 4% formaldehyde and stained with 0.005% crystal violet in phosphate-buffered saline for 1 h.

Cell proliferation assay

Cell proliferation was measured by the CellTiter 96 AQueous Non-Radioactive Cell Proliferation (MTS) assay (Promega Corp.). The cells were cultured in medium for different periods of time. MTS/PMS solution was then added to each well. After 2 h, the absorbances at 490 nm were measured using an EnVision multilabel plate reader (PerkinElmer, Waltham, MA, USA).

Supplementary figure legends:

Supplementary Table 1. Clinical data of patients with lung cancer for the cDNA array samples. cDNA array prepared from the data of patients with lung cancer was purchased from OriGene Technologies, Inc. (MD, USA) for the analysis of USP17 expression and correlation of its expression with the expression of inflammatory and macrophage markers in normal and lung cancer samples. These clinical data were provided by the manufacturer.

Supplementary Table 2. Sequences of forward and reverse primers used for the real-time quantitative polymerase chain reaction analysis of human and mouse genes.

Supplementary Figure 1. Correlation between the expression of ubiquitin-specific peptidase 17 and inflammatory and macrophage markers in lung cancers. Data in Fig. 1c–e were analyzed by the Pearson's correlation coefficient for determining the correlation of the expression of USP17 with (**a**) inflammatory markers and (**b**) macrophage markers in lung cancers.

Supplementary Figure 2. Induction of ubiquitin-specific peptidase 17 expression in lung cancer cells by different cytokines. (**a**) Human H1299 lung cancer cells and (**b**) mouse D121 lung cancer cells were treated with different concentrations of different cytokines for 1 h. Induction of ubiquitin-specific peptidase 17 was analyzed by real-time quantitative polymerase chain reaction. Data represent mean ± standard deviation of three independent experiments, $^{**}P < 0.01$ compared with the time 0 group.

Supplementary Figure 3. Transcription factor binding sites in the promoter region of ubiquitin-specific peptidase 17. A 2-kb promoter region of ubiquitin-specific peptidase 17 (USP17) was analyzed by computer software from the Gene Promoter Miner website (http://gpminer.mbc.nctu.edu.tw/). Key transcription factors that mediate cytokine-induced gene activations, including HIF-1, STAT3, STAT6, and NF-κB, are shown. The arrowhead shows the transcriptional start site of USP17. The nucleotide position of the binding site is shown in parenthesis. The red bar indicates a reverse-strand binding site, and the blue bar indicates a forward-strand binding site.

Supplementary Figure 4. Expression of ubiquitin-specific peptidase 17 in different stable cell lines. Expression levels of ubiquitin-specific peptidase 17 (USP17) in (**a**) H1299, (**b**) D121, and (**c**) LLC cells stably transfected with control, overexpression or shRNA knockdown vector. Expression levels were detected by immunoblotting with specific antibodies as indicated. The blots shown represent a set of three independent blots with similar results.

Supplementary Figure 5. Effect of USP17 on IL-1β-induced NF-κB activation. (**a**) H1299 cells stably overexpressing ubiquitin-specific peptidase 17 (USP17) or stably transfected with control vector were transfected with NF-κB-controlled luciferase reporter gene and treated with or without 10 ng/ml interleukin (IL)-1β. These cells were lysed, and the relative luciferase activities were analyzed. Data represent mean ± standard deviation of three independent experiments, $^{**}P < 0.01$. (**b**) H1299 cells stably overexpressing USP17 or stably transfected with control vector were treated with or without 10 ng/ml IL-1β. The expression of USP17 in these cells was analyzed by immunoblotting with specific antibodies as indicated. The blots shown are representative of three independent experiments..

Supplementary Figure 6. Cell surface expression of stemness markers in ubiquitin-specific peptidase 17 stably overexpressing cells. D121 cells stably transfected with control or ubiquitin-specific peptidase 17 overexpression vector were analyzed by flow cytometry for the percentage of cells with detectable CD117 and CD133 cancer stem cell markers on the cell surface. Data represent mean ± standard deviation of three independent analyses, $^{**}P < 0.01$ compared with control.

Supplementary Figure 7. Ubiquitin-specific peptidase 17 expression enhances the proliferation and anchorage-independent growth of lung cancer cells. (**a, b)** Ubiquitin-specific peptidase 17 was stably overexpressed in human H1299 and mouse D121 lung cancer cells, and their abilities to undergo cell proliferation (a) and anchorage-independent colony formation (b) were analyzed. Top panels of b indicate representative plates showing colony formation. Bottom bar figures of b indicate enumeration of colonies. Data represent mean ± standard deviation of three independent experiments, $^{*}P < 0.05$; $^{**}P < 0.01$ compared with the time 0 group (a) and the control group (b).

Supplementary Figure 8. Interaction between ubiquitin-specific peptidase 17 and TNFR-associated factor 2 and TNFR-associated factor 3 in Lewis lung cancer cells. (**a**) To determine the endogenous interaction, the endogenous ubiquitin-specific peptidase 17 (USP17) was immunoprecipitated from lysates of Lewis lung cancer (LLC) cells with anti-USP17 antibody. Immunoprecipitates (left panels) and original cell lysates (right panels) were subjected to immunoblotting using anti-TNFR-associated factor 2, anti-TNFR-associated factor 3, and anti-USP17 antibody as indicated. The blots shown are representative of three independent experiments. (**b**) To determine the requirement of TRAF2 and TRAF3 binding for regulating nuclear factor-κB (NF-κB) activation by USP17. Lewis lung cancer cells were cotransfected with an NF-κB-controlled luciferase-reporter plasmid and expression vectors encoding wt USP17 and different USP17 mutants as indicated. These cells were treated with or without interleukin (IL)-1β (10 ng/ml), and the relative luciferase activities were analyzed. (**c**) To

determine the requirement of deubiquitinase activity of USP17 for regulating NF-κB activation, LLC cells were cotransfected with an NF-κB-controlled luciferase-reporter plasmid and expression vectors encoding wt USP17 and C89S USP17. These cells were treated with or without IL-1β (10 ng/ml), and the relative luciferase activities were analyzed. The data in b and c represent mean ± standard deviation of three independent experiments, $^{**}P < 0.01$.

Supplementary Figure 9. Efficiency of clodronate-containing liposomes in the depletion of mouse macrophages. C57BL/6J mice were injected with 200 μl of clodronate-containing liposomes and blood samples were collected on the next day. The whole blood cells were incubated with APC-conjugated anti-CD45 antibody for leukocytes and PE-conjugated anti-F4/80 antibody for macrophages. The population of macrophages in blood cells was analyzed by flow cytometry. The histograms shown are representative of three independent experiments. Bar figure, the data represent mean ± standard deviation (n = 3), $^{**}P < 0.01$.

Supplementary table 1

Number	Gender	Age	Tissue of (Origin/Finding)	Appearance	SAMPLE diagnosis from pathology verification	CASE diagnosis from Donor Institution pathology report	Tumor Grade	Minimum Stage Grouping
1	F	67	Lung / Lung	Normal	Within normal limits	Carcinoma of lung, squamous cell	Not Applicable	Not Applicable
2	M	67	Lung / Lung	Normal	Within normal limits	Carcinoma of lung, squamous cell	Not Applicable	Not Applicable
3	F	74	Lung / Lung	Normal	Within normal limits	Adenocarcinoma of lung, bronchioloalveolar	Not Applicable	Not Applicable
4	M	58	Lung / Lung	Normal	Within normal limits	Carcinoma of lung, large cell, neuroendocrine	Not Applicable	Not Applicable
5	M	44	Lung / Lung	Normal	Within normal limits	Carcinoma of lung, squamous cell	Not Applicable	Not Applicable
6	M	61	Lung / Lung	Normal	Within normal limits	Adenocarcinoma of lung	Not Applicable	Not Applicable
7	M	45	Lung / Lung	Normal	Within normal limits	Malignant melanoma, metastatic	Not Applicable	Not Applicable
8	M	68	Lung / Lung	Tumor	Carcinoma of lung, squamous cell	Carcinoma of lung, squamous cell	Not Reported	I
9	M	69	Lung / Lung	Tumor	Adenocarcinoma of lung	Adenocarcinoma of lung	AJCC G2: Moderately differentiated	I
10	M	51	Lung / Lung	Tumor	Adenocarcinoma of lung, papillary	Adenocarcinoma of lung, papillary	Not Reported	I
11	M	62	Lung / Lung	Tumor	Carcinoma of lung, squamous cell	Carcinoma of lung, squamous cell	AJCC G2: Moderately differentiated	I
12	M	63	Lung / Lung	Tumor	Carcinoma of lung, large cell	Carcinoma of lung, large cell	AJCC G3: Poorly differentiated	I
13	F	45	Lung / Lung	Tumor	Adenocarcinoma of lung, mucinous	Adenocarcinoma of lung, mucinous	AJCC G2: Moderately differentiated	I
14	F	38	Lung / Lung	Tumor	Carcinoma of lung, squamous cell	Carcinoma of lung, squamous cell	Not Reported	I
15	M	74	Lung / Lung	Tumor	Adenocarcinoma of lung	Adenocarcinoma of lung	AJCC G3: Poorly differentiated	I
16	F	62	Lung / Lung	Tumor	Adenocarcinoma of lung	Adenocarcinoma of lung	AJCC G1: Well differentiated	I
17	F	66	Lung / Lung	Tumor	Adenocarcinoma of lung	Adenocarcinoma of lung	AJCC G2: Moderately differentiated	I
18	F	81	Lung / Lung	Tumor	Adenocarcinoma of lung, bronchioloalveolar	Adenocarcinoma of lung, bronchioloalveolar	AJCC G1: Well differentiated	I
19	M	54	Lung / Lung	Tumor	Adenocarcinoma of lung	Adenocarcinoma of lung	AJCC G2: Moderately differentiated	II
20	M	78	Lung: left lower lobe / Lung: left lower lobe	Tumor	Carcinoma of lung, large cell, neuroendocrine	Carcinoma of lung, large cell, neuroendocrine	AJCC G3: Poorly differentiated	II
21	F	84	Lung: right upper lobe / Lung: right upper lobe	Tumor	Adenocarcinoma of lung	Adenocarcinoma of lung	AJCC G2: Moderately differentiated	II
22	M	77	Lung: left lower lobe / Lung: left lower lobe	Tumor	Adenocarcinoma of lung	Adenocarcinoma of lung	AJCC G3: Poorly differentiated	II
23	F	67	Lung / Lung	Tumor	Carcinoma of lung, squamous cell	Carcinoma of lung, squamous cell	AJCC G3: Poorly differentiated	II
24	M	80	Lung / Lung	Tumor	Carcinoma of lung, squamous cell	Carcinoma of lung, squamous cell	AJCC G3: Poorly differentiated	II
25	M	69	Lung / Lung	Tumor	Carcinoma of lung, squamous cell	Carcinoma of lung, squamous cell	AJCC G3: Poorly differentiated	II
26	M	51	Lung / Lung	Tumor	Carcinoma of lung, squamous cell	Carcinoma of lung, squamous cell	Not Reported	II
27	F	77	Lung / Lung	Tumor	Carcinoma of lung, squamous cell	Carcinoma of lung, squamous cell	AJCC G2: Moderately differentiated	II
28	F	68	Lung / Lung	Tumor	Adenocarcinoma of lung	Adenocarcinoma of lung	AJCC G3: Poorly differentiated	II
29	F	51	Lung / Lung	Tumor	Carcinoma of lung, squamous cell	Carcinoma of lung, squamous cell	AJCC G2: Moderately differentiated	II
30	M	75	Lung: right upper lobe / Lung: right upper lobe	Tumor	Adenocarcinoma of lung	Adenocarcinoma of lung	AJCC G3: Poorly differentiated	II
31	F	77	Lung / Lung	Tumor	Adenocarcinoma of lung	Adenocarcinoma of lung	AJCC G2: Moderately differentiated	II
32	F	73	Lung / Lung	Tumor	Carcinoma of lung, non-small cell	Carcinoma of lung, non-small cell	AJCC G3: Poorly differentiated	III
33	M	47	Lung / Lung	Tumor	Adenocarcinoma of lung	Adenocarcinoma of lung	Not Reported	III
34	F	68	Lung: right upper lobe / Lung: right upper lobe	Tumor	Adenocarcinoma of lung	Adenocarcinoma of lung, acinar	Not Reported	III
35	F	72	Lung / Lung	Tumor	Adenocarcinoma of lung	Adenocarcinoma of lung	AJCC G3: Poorly differentiated	III
36	M	55	Lung / Lung	Tumor	Adenocarcinoma of lung	Adenocarcinoma of lung	AJCC G3: Poorly differentiated	III
37	F	64	Lung: left lower lobe / Lung: left lower lobe	Tumor	Adenocarcinoma of lung	Adenocarcinoma of lung	AJCC G2: Moderately differentiated	III
38	M	65	Lung: left lower lobe / Lung: left lower lobe	Tumor	Carcinoma of lung, squamous cell	Carcinoma of lung, squamous cell	AJCC G2: Moderately differentiated	III
39	F	73	Lung / Lung	Tumor	Carcinoma of lung, squamous cell	Carcinoma of lung, squamous cell	AJCC G3: Poorly differentiated	III
40	M	52	Lung / Lung	Tumor	Carcinoma of lung, adenosquamous	Carcinoma of lung, adenosquamous	AJCC G3: Poorly differentiated	III
41	M	61	Lung: left upper lobe / Lung: left upper lobe	Tumor	Carcinoma of lung, small cell	Carcinoma of lung	AJCC G3: Poorly differentiated	III
42	M	72	Lung: left lower lobe / Lung: left lower lobe	Tumor	Carcinoma of lung, squamous cell	Carcinoma of lung, squamous cell	AJCC G2: Moderately differentiated	III
43	M	51	Lung / Lung	Tumor	Adenocarcinoma of lung	Adenocarcinoma of lung	AJCC G2: Moderately differentiated	III
44	F	65	Lung / Lung	Tumor	Adenocarcinoma of lung, bronchioloalveolar	Adenocarcinoma of lung	AJCC G1: Well differentiated	III
45	M	63	Lung / Lymph node	Tumor	Carcinoma of lung, non-small cell, metastatic	Carcinoma of lung, non-small cell, metastatic	AJCC G3: Poorly differentiated	III
46	M	56	Lung / Lung	Tumor	Adenocarcinoma of lung	Adenocarcinoma of lung	AJCC G3: Poorly differentiated	IV
47	M	77	Lung / Lung	Tumor	Carcinoma of lung, squamous cell	Carcinoma of lung, squamous cell	AJCC G3: Poorly differentiated	IV
48	M	53	Lung / Brain	Tumor	Adenocarcinoma of lung, metastatic	Adenocarcinoma of lung, metastatic	Not Reported	IV

Human	forward	reverse
TNF-α	AACCTCCTCTCTGCCATCAA	CCAAAGTAGACCTGCCCAGA
IL-1β	ACGATGCACCTGTACGATCA	TCTTTCAACACGCAGGACAG
IL-6	TACCCCCAGGAGAAGATTCC	TTTTCTGCCAGTGCCTCTTT
IL-8	GTGCAGTTTTGCCAAGGAGT	CTCTGCACCCAGTTTTCCTT
IL-12A	ACTAGAGAGACTTCTTCCACAACAAGAG	GCACAGGGTCATCATCAAAGAC
IL-23A	GCAGATTCCAAGCCTCAGTC	CCTTGAGCTGCTGCCTTTAG
CCL2	CCCCAGTCACCTGCTGTTAT	TGGAATCCTGAACCCACTTC
CCL13	CTCCTCTGGCCTCCTCTTCT	ACCGAATACAAACCCACTGC
CCL18	TGCCCAGCCACATTAACTAAC	GGCACAATGTCTGCTGAGAA
CCL19	GGTGCCTGCTGTAGTGTTCA	GGTCCTTCCTTCTGGTCCTC
CCL20	GCGCAAATCCAAAACAGACT	CAAGTCCAGTGAGGCACAAA
CXCL11	TCGAAGCAAGCAAGGCTTAT	GTCCTTTCACCCACCTTTCA
CCR7	ACATCGGAGACAACACCACA	GGAAGGGTCAGGAGGAAGAG
NOS2	ACAAGCCTACCCCTCCAGAT	TCCCGTCAGTTGGTAGGTTC
MMP9	CTCGAACTTTGACAGCGACA	GCCATTCACGTCGTCCTTAT
INDO	GATGAAGAAGTGGGCTTTGC	CGGACATCTCCATGACCTTT
MRC1	TGACACACTTTTGGGGATCA	AAACTTGAACGGGAATGCAC
MAF	AGAGACACGTCCTGGAGTCG	GCTTCCAAAATGTGGCGTAT
ARG1	GGCTGGTCTGCTTGAGAAAC	TTCCCACAGACCTTGGATTC
USP17	CAAACATGTCAGCGTCCCAA	ATTTGGTGGATGAGGGTGGT
GADPH	GAGTCAACGGATTTGGTCGT	GACAAGCTTCCCGTTCTCAG
FLG2	AGGTCCAGCTGTGGTCATTC	TGCCCACTAGTCTCCAATCC
c-MYC	TTCGGGTAGTGGAAAACCAG	CAGCAGCTCGAATTTCTTCC
SOX-2	ACACCAATCCCATCCACACT	GCAAACTTCCTGCAAAGCTC
OCT-4	GAAGGATGTGGTCCGAGTGT	GTGAAGTGAGGGCTCCCATA
KLF-4	ACCCACACAGGTGAGAAACC	ATGTGTAAGGCGAGGTGGTC
NANOG	TTCCTTCCTCCATGGATCTG	ATCTGCTGGAGGCTGAGGTA
ALDH1	TGTTAGCTGATGCCGACTTG	CTTCTTAGCCCGCTCAACAC
ABCG2	GTGGCCTTGGCTTGTATGAT	AACAATTGCTGCTGTGCAAC
CD44	AAGGTGGAGCAAACACAACC	AGCTTTTTCTTCTGCCCACA
CD117	AGAGACTTGGCAGCCAGAAA	AGGGGCTGCTTCCTAAAGAG
CD133	GCCACCGCTCTAGATACTGC	TGTTGTGATGGGCTTGTCAT

Mouse	forward	reverse
TNF-α	AGCCCCCAGTCTGTATCCTT	CTCCCTTTGCAGAACTCAGG
IL-1β	CAGGCAGGCAGTATCACTCA	AGCTCATATGGGTCCGACAG
IL-6	AGTTGCCTTCTTGGGACTGA	TCCACGATTTCCCAGAGAAC
IL-8	CGTCCCTGTGACACTCAAGA	TAATTGGGCCAACAGTAGCC
IL-12A	CTCCTGTGGGAGAAGCAGAC	CAGATAGCCCATCACCCTGT
IL-23A	AATAATGTGCCCCGTATCCA	CATGGGGCTATCAGGGAGTA
CCL2	CAGGTCCCTGTCATGCTTCT	TCTGGACCCATTCCTTCTTG
CCL18	GCAGTCTTTGAGGTGGAAGC	CATCCATCCAAGATGTGCAG
CCL19	TTCCCAGCGGATTTTAAGTG	GCAAAAGAGGCAGACAGACC
CCL20	CTTGCTTTGGCATGGGTACT	AGCCCTTTTCACCCAGTTCT
CXCL11	CAGTGCTGGATTCAAAAGCA	AACCCCTTAGAAGGCCTCAG
CCR7	TGTACGAGTCGGTGTGCTTC	TAGGCCCAGAAGGGAAGAAT
NOS2	CACCTTGGAGTTCACCCAGT	ACCACTCGTACTTGGGATGC
MMP9	GAAGGCAAACCCTGTGTGTT	AGAGTACTGCTTGCCCAGGA
INDO	AAGGGCTTCTTCCTCGTCTC	GCATTTCCAGCCAGACAGAT
MRC1	TGGCAAGTGTCCAGAGTCAG	TCCCTTCAACATTTCGGAAC
MAF	TCCTGAGTGGGCTTGCTAGT	AAGTTACGGGGGAATTCAGG
ARG1	CGCCTTTCTCAAAAGGACAG	GACATCAACAAAGGCCAGGT
USP17	GGTGGGAGATGAAGATCGAA	CGTGAAGGGTAAGGCATTGT
GADPH	ACCCAGAAGACTGTGGATGG	CACATTGGGGGTAGGAACAC
FLG2	CCCAAAGTCCTTTTCATCCA	CCAGAAGGTCCATACCCAGA
MYC	ACACGGAGGAAAACGACAAG	TCGTCTGCTTGAATGGACAG
SOX-2	AAGGGTTCTTGCTGGGTTTT	AGACCACGAAAACGGTCTTG
OCT-4	AAGCCCTCCCTACAGCAGAT	CTGGGAAAGGTGTCCCTGTA
KLF-4	GCAGTCACAAGTCCCCTCTC	CTGTGTGAGTTCGCAGGTGT
NANOG	CCAGTGGAGTATCCCAGCAT	GAAGTTATGGAGCGGAGCAG
ALDH1	GCACTCAATGGTGGGAAAGT	TTTGGCCACACACTCCAATA
ABCG2 -	CCATTCATCAGCCTCGGTAT	AATCCGCAGGGTTGTTGTAG
CD44	TGGATCCGAATTAGCTGGAC	AGCTTTTTCTTCTGCCCACA
CD117	GGGCTAGCCAGAGACATCAG	AGGAGAAGAGCTCCCAGAGG
CD133	GAAAAGTTGCTCTGCGAACC	TCTCAAGCTGAAAAGCAGCA

Supplementary figure 1

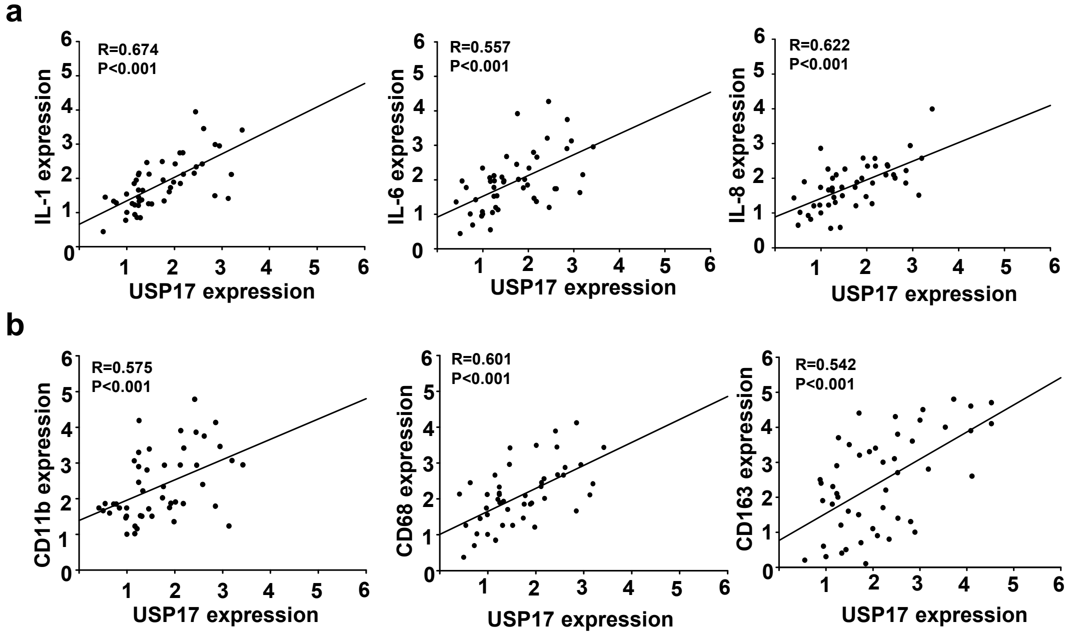

Supplementary figure 2

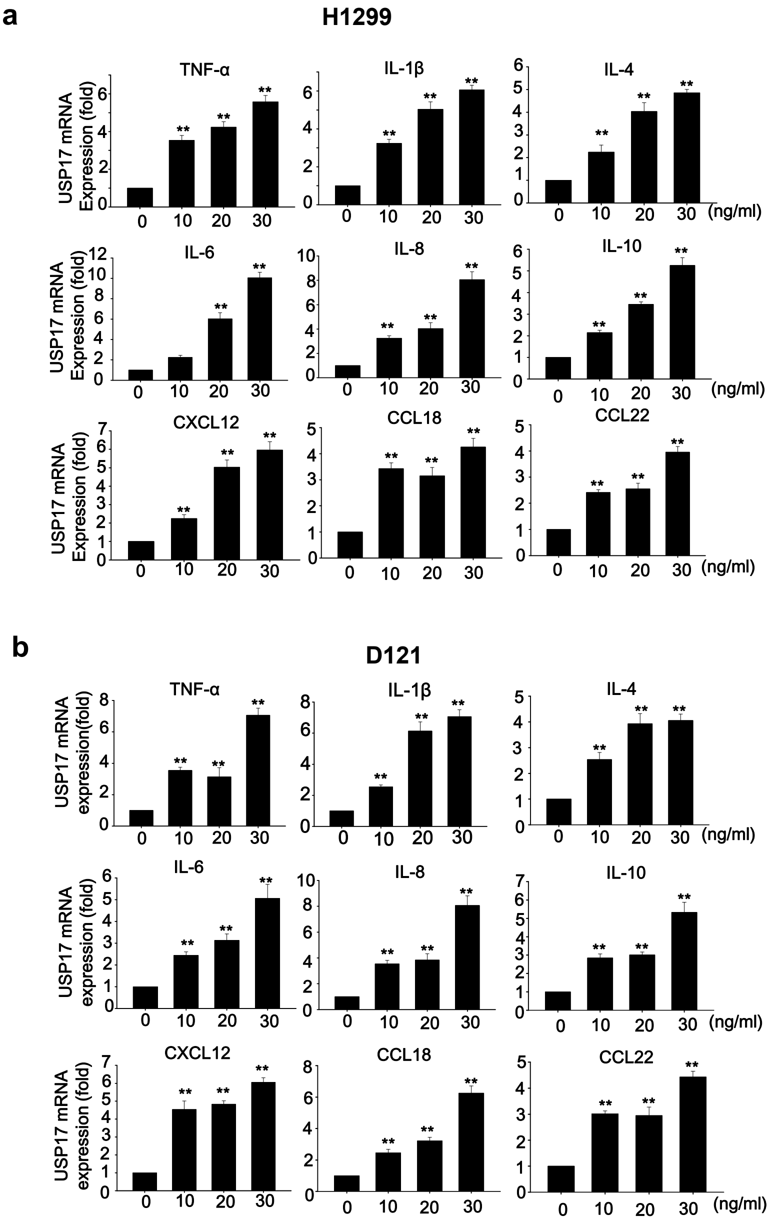

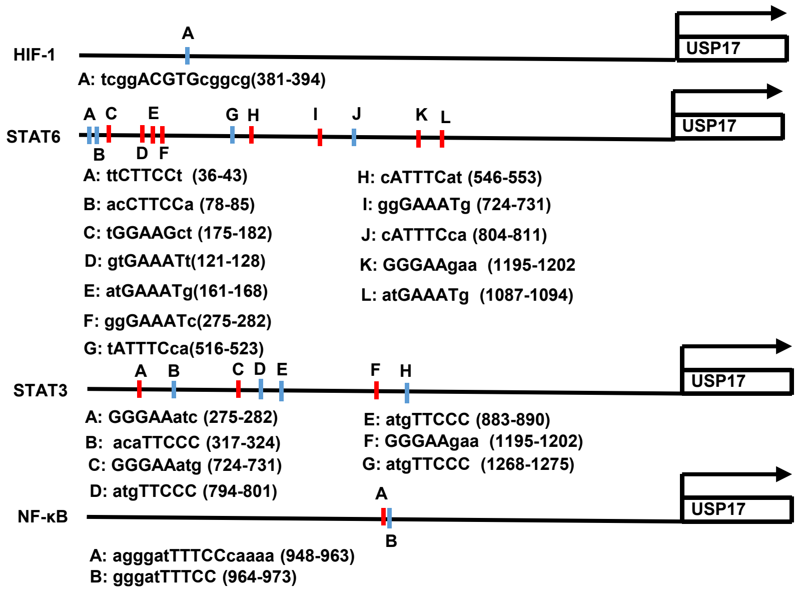
HIF-1
A
USP17
A: tcggACGTGcggcg(381-394)
STAT6
A C E G H I J K L
B D F
USP17
A: ttCTTCCt (36-43)
B: acCTTCCa (78-85)
C: tGGAAGct (175-182)
D: gtGAAATt(121-128)
E: atGAAATg(161-168)
F: ggGAAATc(275-282)
G: tATTTCca(516-523)
H: cATTTCat (546-553)
I: ggGAAATg (724-731)
J: cATTTCca (804-811)
K: GGGAAgaa (1195-1202
L: atGAAATg (1087-1094)
STAT3
A B C D E F H
USP17
A: GGGAAatc (275-282)
B: acaTTCCC (317-324)
C: GGGAAatg (724-731)
D: atgTTCCC (794-801)
E: atgTTCCC (883-890)
F: GGGAAgaa (1195-1202)
G: atgTTCCC (1268-1275)
NF-κB
A
B
USP17
A: agggatTTTCCcaaaa (948-963)
B: gggatTTTCC (964-973)

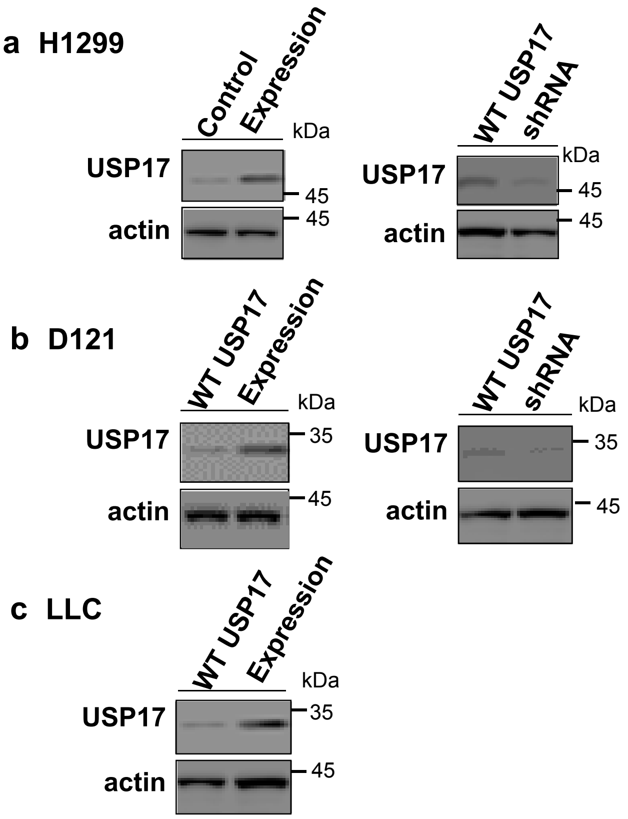
a H1299
Control
Expression
kDa
USP17
45
actin
45
WT USP17
shRNA
kDa
USP17
45
actin
45
b D121
WT USP17
Expression
kDa
USP17
35
actin
45
WT USP17
shRNA
kDa
USP17
35
actin
45
c LLC
WT USP17
Expression
kDa
USP17
35
actin
45

a

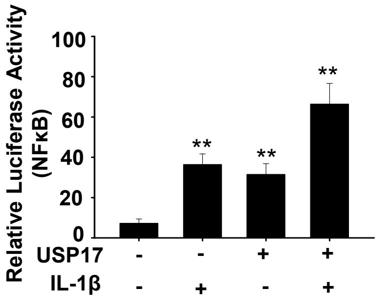
Relative Luciferase Activity (NFκB)
100
80
60
40
20
0
**
**
**
USP17 - - + +
IL-1β - + - +

b

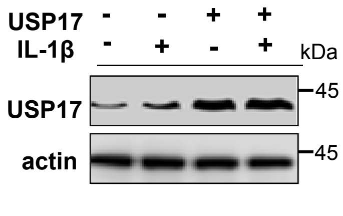
USP17 - - + +
IL-1β - + - +
kDa
USP17
actin
45
45

Supplementary figure 6

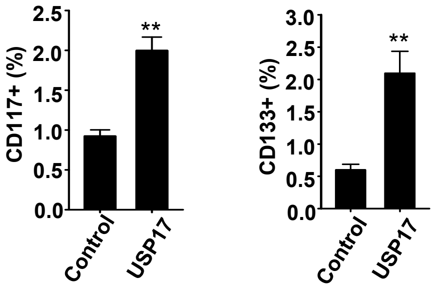

Supplementary figure 7

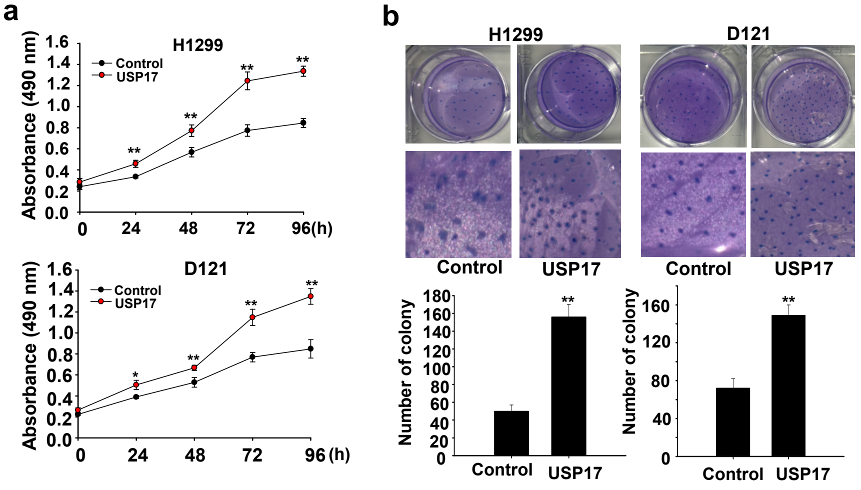

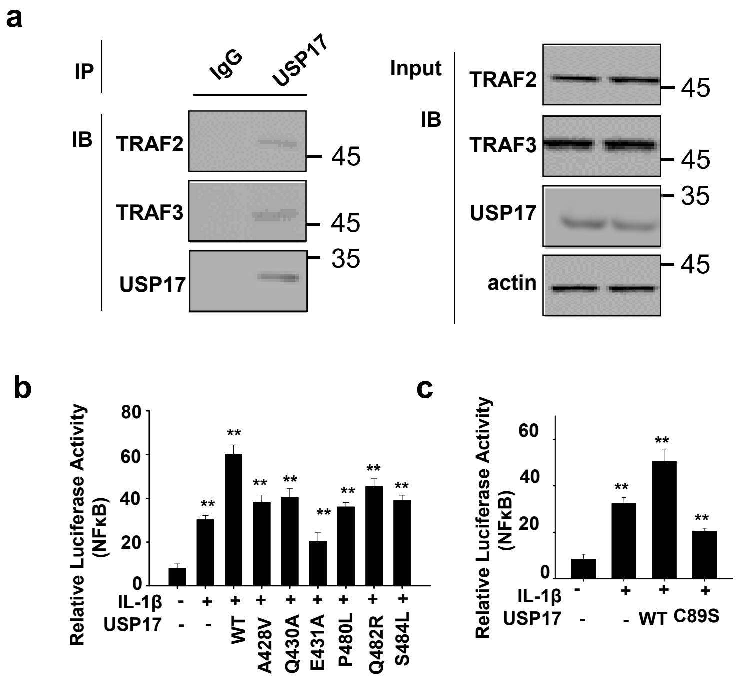
a
IP
IgG
USP17
IB
TRAF2
45
TRAF3
45
35
USP17
Input
TRAF2
45
IB
TRAF3
45
35
USP17
45
actin
b
Relative Luciferase Activity (NFκB)
80
60
40
20
0
IL-1β
USP17
WT
A428V
Q430A
E431A
P480L
Q482R
S484L
c
Relative Luciferase Activity (NFκB)
60
40
20
0
IL-1β
USP17
WT
C89S

Supplementary figure 9

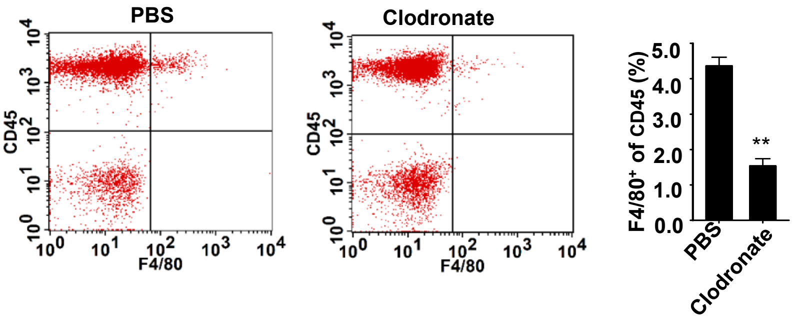

Supplement: Supplementary file 1 — Supplementary Information [file 41388_2018_411_MOESM1_ESM.pdf]
